# Supplementary material for: The Diagnostic Value of EEG Wave Trains for Distinguishing Immature Absence Seizures and Sleep Spindles: Evidence from the WAG/Rij Rat Model
Source: Diagnostics (Basel). 2025 Apr 12;15(8):983. doi: 10.3390/diagnostics15080983 (PMC12025834; doi:10.3390/diagnostics15080983)
Supplement: Supplementary file 1 [file diagnostics-15-00983-s001.zip › diagnostics-3544622-supplementary.pdf]

Supplementary Materials: The Diagnostic Value of EEG Wave Trains for Distinguishing Immature Absence Seizures and Sleep Spindles: Evidence from the WAG/Rij Rat Model

Olga S. Sushkova, Alexei A. Morozov, Alexandra V. Gabova, Karine Yu. Sarkisova

Section S1

Figure S1 demonstrates slices of the wavelet spectrogram of an EEG wave train in the cerebral cortex of a rat.

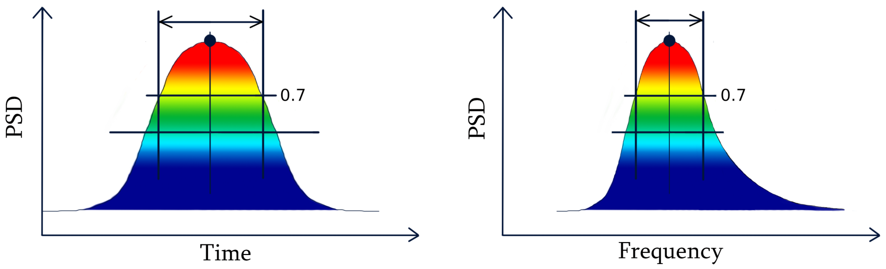

Figure S1. Two slices of the wavelet spectrogram of an EEG wave train in the cerebral cortex of a rat. In the left: a time slice of the wavelet spectrogram. Arrows indicate the duration of the wave train measured at $1/\sqrt{2}$ of full height. The abscissa axis indicates the time. The ordinate indicates PSD. In the right: a frequency slice of the wavelet spectrogram. Arrows indicate the bandwidth of the wave train measured at $1/\sqrt{2}$ of full height. The abscissa axis indicates the frequency. The ordinate indicates PSD.

Let M be a local maximum in the spectrogram. From this point, we drop a perpendicular to the time-frequency plane. At a height of $1/\sqrt{2}$ from the full height, we draw perpendiculars parallel to the frequency and time axes. This is the width of the wave train in time and frequency. The width in time and the width in frequency form a quadrangle (Figure S2). Note that the width can also be measured at a different height. The height $1/\sqrt{2}$ was chosen experimentally by comparing several threshold values that allow the best detection of the wave trains.

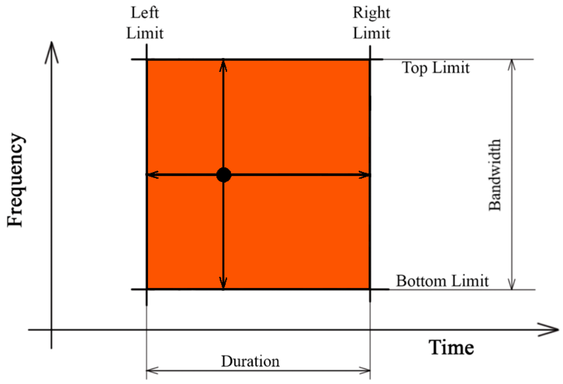

Figure S2. Boundaries of the wave train in the EEG wavelet spectrogram.

Then we check whether there are no values greater than the value of the considered local maximum M in the considered rectangular region. We consider the maximum M as

a wave train of the electrical activity of the brain if the duration W_{Time} of the wave train M is greater than or equal to the established threshold T. The threshold T is a function depending on the frequency f of local maximum M (1,2):

$$T = \frac{NP}{f} \tag{1}$$

$$W_{Time} \geq T \tag{2}$$

where NP is the minimum number of periods in a wave train, a constant set by an expert. In this paper, $NP = 0.1$ was used.

Section S2

ROC curves (receiver operating characteristic) and AUC (area under the ROC curve) compare the number of wave trains in wavelet spectrograms observed in datasets. Let us consider two sets A and B of the number of wave trains corresponding to different EEG fragments. Set A corresponds to immature discharges (9 fragments) and set B corresponds to background EEG (9 fragments). The power of each set is equal to the number of EEG fragments in the corresponding dataset. The ROC curve is calculated as follows. In a loop, various values of the threshold for the number of wave trains are sorted out from the smallest value in sets A and B to the largest value in these sets. For each threshold value, it is checked whether this threshold can separate the number of wave trains in immature discharges and background EEG. Four vectors are calculated based on the result of considering all threshold values. Vector TP is the number of elements of set A that exceed the threshold value. Vector TN is the number of elements of set B that do not exceed the threshold value. Vector FP is the number of elements of set B that exceed the threshold value. Vector FN is the number of elements of set A that do not exceed the threshold value. The vector of false positive rates $FPR = FP/(TN + FP)$ and the vector of true positive rates $TPR = TP/(TP + FN)$ are calculated. A ROC curve is constructed based on the calculated vectors FPR and TPR; the abscissa axis indicates the false positive rate (FPR) and the ordinate axis indicates the true positive rate (TPR). The area under the ROC curve (AUC) is an indicator of the magnitude of the difference between the number of wave trains in immature discharges and the background EEG. When constructing a frequency AUC diagram, the calculation of the ROC curve and AUC value is repeated many times for different frequency ranges of the EEG signals.

Section S3

In Figure S3, a PSD AUC diagram comparing immature discharges with the background EEG is demonstrated. The PSD AUC diagram confirms the results obtained using the genetic optimization algorithm, namely, the presence of a pronounced boundary between PSD ranges characteristic of immature discharges and the background EEG in the area of 50 000–60 000 $\mu V^2/Hz$.

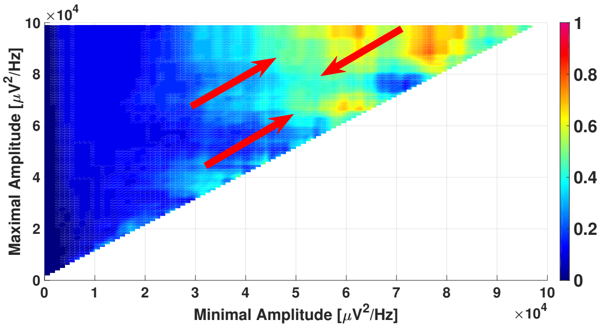

Figure S3. A PSD AUC diagram compares immature discharges (9) with the background EEG (9). The abscissa axis indicates the lower limit of the PSD range; the ordinate axis indicates the upper limit of the PSD range. The diagram demonstrates a pronounced boundary between the ranges of PSD characteristic of immature discharges and background EEG; the boundary is indicated by red arrows.

Section S4

In Figure S4, a bandwidth AUC diagram is demonstrated comparing immature discharges with the background EEG. The bandwidth AUC diagram confirms the results obtained using the genetic optimization algorithm, namely, the presence of a pronounced boundary between the wave train bandwidth characteristic of immature discharges and the background EEG in the area of 2–5 Hz.

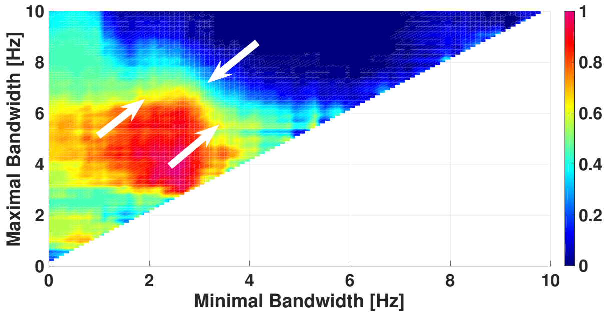

Figure S4. A bandwidth AUC diagram compares immature discharges (9) with the background EEG (9). The abscissa axis indicates the lower limit of the bandwidth; the ordinate axis indicates the upper limit of the bandwidth. The diagram demonstrates a pronounced boundary between the bandwidth of wave trains characteristic of immature discharges and the background EEG. The boundary is indicated by white arrows.

Section S5

In Figure S5, a duration in periods AUC diagram is demonstrated comparing immature discharges with sleep spindles. The AUC diagram confirms the results obtained by the genetic optimization algorithm, namely, the presence of a pronounced boundary between the ranges of wave train duration in periods characteristic of immature discharges and sleep spindles in the area of 1.6–2.1 periods.

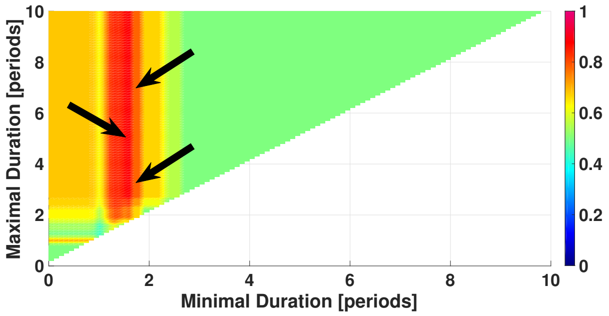

Figure S5. A duration in periods AUC diagram compares immature discharges (9) with sleep spindles (20). The diagram is constructed using the following constraints on wave train parameters: the central frequency is no more than 8.5 Hz, the bandwidth is no less than 1.7 Hz, and the instantaneous phase is from -3.14 to +1.5 radians. The abscissa axis indicates the lower limit of the duration range in periods; the ordinate axis indicates the upper limit of the duration range in periods. The diagram demonstrates a pronounced boundary between the duration ranges characteristic of immature discharges and sleep spindles. The boundary is indicated by black arrows.

In Figure S6, a bandwidth AUC diagram is demonstrated comparing immature discharges with sleep spindles. The AUC diagram confirms the results obtained by the genetic optimization algorithm, namely, the presence of a pronounced boundary between the ranges of bandwidth characteristic of immature discharges and sleep spindles in the area 1.5–2 Hz.

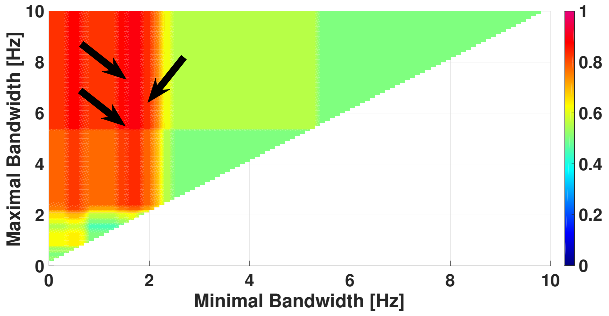

Figure S6. A bandwidth AUC diagram compares immature discharges (9) with sleep spindles (20). The diagram is constructed using the following constraints on wave train parameters: the central frequency is no more than 8.5 Hz, the wave train duration in periods is no less than 1.6 periods, and the instantaneous phase is from -3.14 to +1.5 radians. The abscissa axis indicates the lower limit of the bandwidth; the ordinate axis indicates the upper limit of the bandwidth. The diagram demonstrates a pronounced boundary between the bandwidth characteristic of immature discharges and the bandwidth characteristic of sleep spindles. The boundary is indicated by black arrows.

In Figure S7, an instantaneous phase AUC diagram is demonstrated comparing immature discharges with sleep spindles. Note that the instantaneous phase AUC diagram includes an additional triangular region at the bottom (under the diagonal line). The meaning of this region is that the coordinates of the points in this area correspond to the ranges of instantaneous phase excluded from consideration when comparing data sets. The AUC diagram confirms the results obtained by the genetic optimization algorithm, namely, the presence of a pronounced boundary between the instantaneous phase ranges characteristic of immature discharges and sleep spindles in the area of 1.5–2 radians.

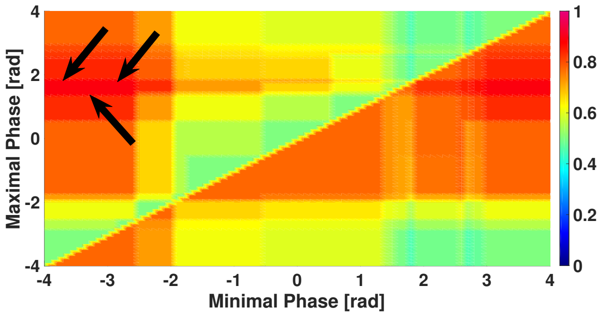

Figure S7. An instantaneous phase AUC diagram compares immature discharges (9) with sleep spindles (20). The diagram is constructed using the following constraints on the wave train parameters: the central frequency is no more than 8.5 Hz, the wave train duration in periods is no less than 1.6 periods, and the frequency bandwidth is no less than 1.7 Hz. The abscissa axis indicates the lower boundary of the instantaneous phase; the ordinate axis indicates the upper boundary of the instantaneous phase. The diagram demonstrates a pronounced boundary between the ranges of the instantaneous phase characteristic of immature discharges and sleep spindles. The boundary is indicated by black arrows.

Section S6

In Figure S8, a bandwidth AUC diagram is demonstrated comparing immature discharges with sleep spindles. The bandwidth AUC diagram confirms the results found by the genetic optimization algorithm, namely, the presence of a pronounced boundary between the wave train bandwidths characteristic of immature discharges and sleep spindles in the area of 4 Hz.

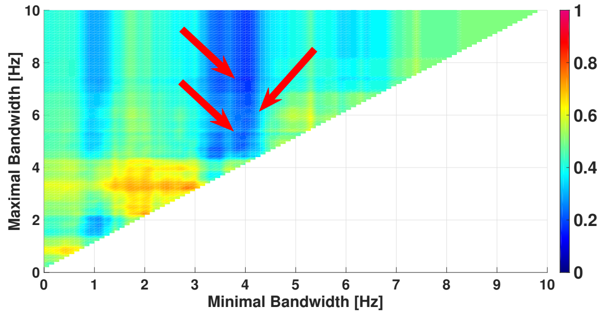

Figure S8. A bandwidth AUC diagram compares immature discharges (9) with sleep spindles (20). The diagram is constructed using the following constraints on the wave train parameters: the central frequency is no more than 15 Hz and the instantaneous phase is from -2.5 to +2.5 radians. The abscissa axis indicates the lower limit of the frequency band; the ordinate axis indicates the upper limit of the frequency band. The diagram demonstrates a pronounced boundary between the bandwidth of wave trains characteristic of immature discharges and sleep spindles. The boundary is indicated by red arrows.

Figure S9 demonstrates an instantaneous phase AUC diagram comparing immature discharges with sleep spindles. The instantaneous phase AUC diagram includes an additional triangular area under the diagonal line. The AUC diagram confirms the results obtained by the genetic optimization algorithm, namely, the presence of an instantaneous phase range typical for sleep spindles and not typical for immature discharges from about -2.5 to +2.5 radians with an AUC of about 0.17.

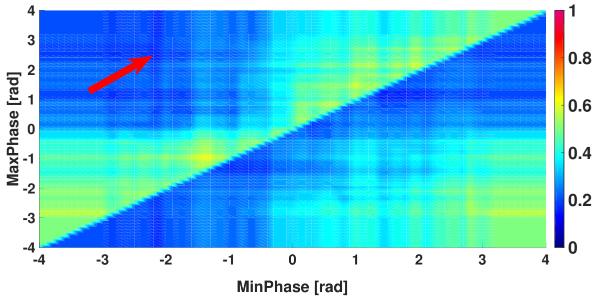

Figure S9. An instantaneous phase AUC diagram compares immature discharges (9) with sleep spindles (20). The diagram is constructed using the following constraints on the wave train parameters: the central frequency is no more than 15 Hz and the frequency bandwidth is no less than 4 Hz. The abscissa axis indicates the lower boundary of the instantaneous phase; the ordinate axis indicates the upper boundary of the instantaneous phase. The diagram demonstrates an instantaneous phase range typical for sleep spindles and not typical for immature discharges from about -2.5 to +2.5 radians with an AUC of about 0.17 (indicated by the red arrow).

Section S7

In Figure S10, an example of a wave train in the wavelet spectrogram corresponding to the red area in the frequency AUC diagram comparing immature discharges (9) with the background EEG (9) is demonstrated.

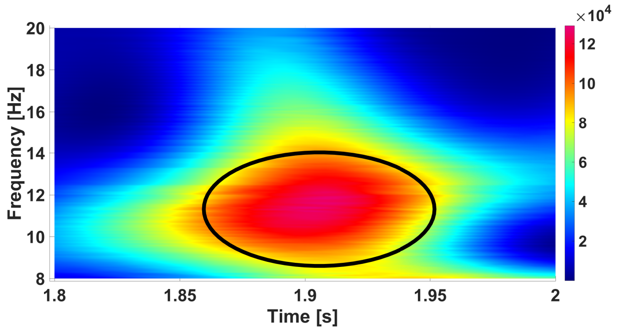

Figure S10. An example of a wavelet spectrogram of an EEG wave train in the cerebral cortex of a rat. The abscissa axis indicates the time in seconds. The ordinate axis indicates the frequency in Hz. The wave train is marked with a black ellipse.

Figure S11 demonstrates a fragment of EEG signal corresponding to the wave train in the wavelet spectrogram in Figure S10.

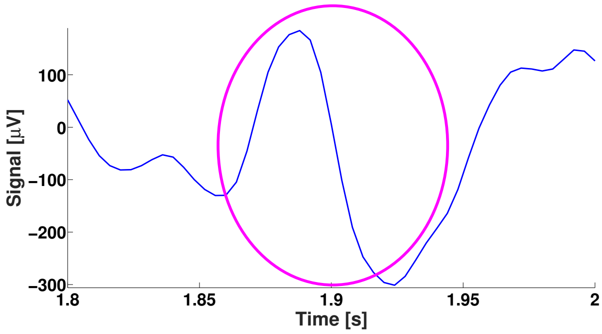

Figure S11. An example of an EEG wave train in the rat cerebral cortex corresponds to the wave train in the wavelet spectrogram in Figure S10. The abscissa axis indicates the time in seconds. The ordinate axis indicates the amplitude in μV. The wave train is marked with a purple ellipse.

Figure S12 demonstrates an example of a wave train in the wavelet spectrogram corresponding to the blue area in the frequency AUC diagram comparing immature discharges (9) with the background EEG (9).

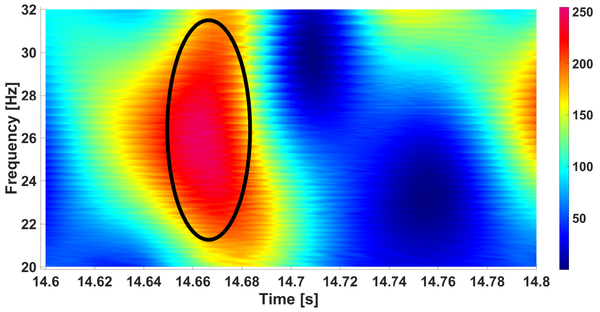

Figure S12. An example of a wave train in a wavelet spectrogram. The wave train parameters correspond to the blue area in the frequency AUC diagram comparing immature discharges (9) with the background EEG (9). The abscissa axis indicates the time in seconds. The ordinate axis indicates the frequency in Hz. The wave train is marked with a black ellipse.

Figure S13 demonstrates a fragment of EEG signal corresponding to the wave train on the wavelet spectrogram in Figure S12.

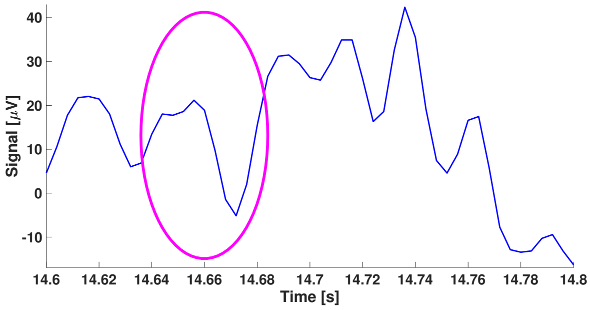

Figure S13. A fragment of EEG signal corresponding to the wave train in the wavelet spectrogram in Figure S12. The wave train parameters correspond to the blue area in the AUC diagrams in the frequency AUC diagram comparing immature discharges (9) with the background EEG (9). The abscissa axis indicates the time in seconds. The ordinate axis indicates the amplitude in μV. The wave train is marked with a purple ellipse.

Note that it is impossible to distinguish the wave trains in Figure S11 and Figure S13 by the waveform. However, using the method of analyzing wave train electrical activity and AUC diagrams, wave train characteristics were determined that allow us to distinguish immature discharges from background EEG.

Section S8

Figure S14 demonstrates an example of a wave train in wavelet spectrograms corresponding to the red area in the frequency AUC diagram comparing immature discharges (9) with sleep spindles (20).

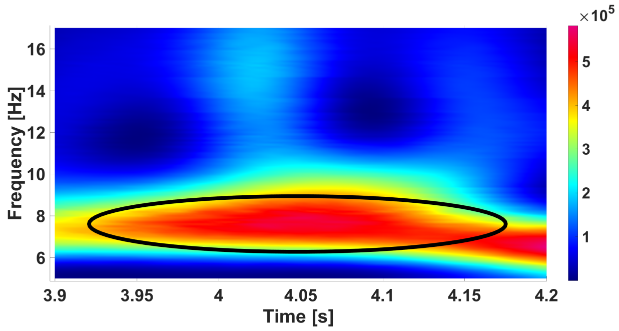

Figure S14. An example of a wave train in a wavelet spectrogram. The wave train parameters correspond to the red area in the AUC diagram comparing immature discharges (9) with sleep spindles (20). The abscissa axis indicates the time in seconds. The ordinate axis indicates the frequency in Hz. The wave train is marked with a black ellipse.

Figure S15 demonstrates a fragment of EEG signal corresponding to the wave train in the wavelet spectrogram in Figure S14.

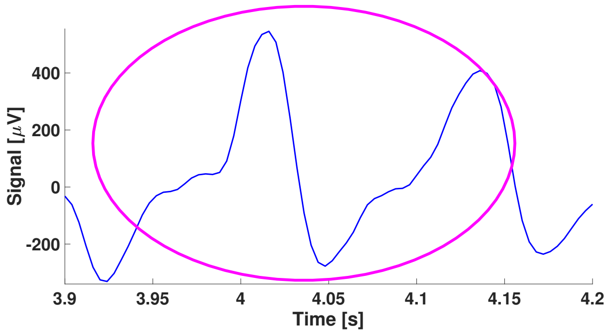

Figure S15. A fragment of the EEG signal corresponding to the wave train in the wavelet spectrogram in Figure S14. The wave train parameters correspond to the red area in the frequency AUC diagram comparing immature discharges (9) with sleep spindles (20). The abscissa axis indicates the time in seconds. The ordinate axis indicates the amplitude in μV. The wave train is marked with a purple ellipse.

Figure S16 demonstrates an example of a wave train in the wavelet spectrogram corresponding to the blue area in the frequency AUC diagram comparing immature discharges (9) with sleep spindles (20).

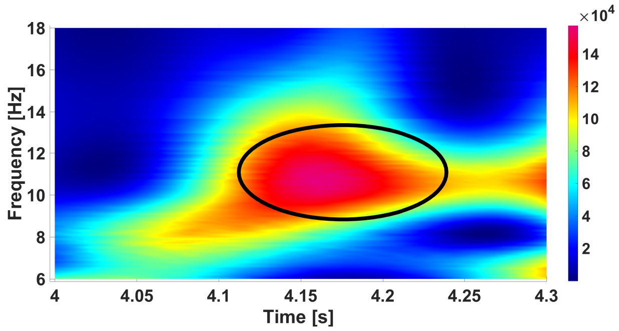

Figure S16. An example of a wave train in a wavelet spectrogram. The wave train parameters correspond to the blue area in the frequency AUC diagram comparing immature discharges (9) with sleep spindles (20). The abscissa axis indicates the time in seconds. The ordinate axis indicates the frequency in Hz. The wave train is marked with a black ellipse.

Figure S17 demonstrates a fragment of EEG signal corresponding to the wave train in the wavelet spectrogram in Figure S16.

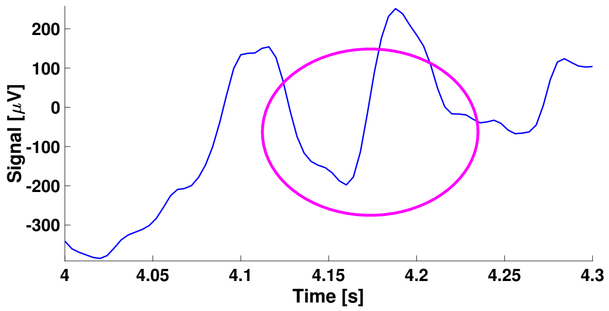

Figure S17. A fragment of EEG signal corresponding to the wave train in the wavelet spectrogram in Figure S16. The wave train parameters correspond to the blue area in the frequency AUC diagram comparing immature discharges (9) with sleep spindles (20). The abscissa axis indicates the time in seconds. The ordinate axis indicates the amplitude in μV. The wave train is marked with a purple ellipse.
